# Supplementary material for: Investigating the influence of physiologically relevant hydrostatic pressure on CHO cell batch culture
Source: Sci Rep. 2021 Jan 8;11:162. doi: 10.1038/s41598-020-80576-8 (PMC7794228; doi:10.1038/s41598-020-80576-8)
Supplement: Supplementary file 1 — Supplementary Information 1. [file 41598_2020_80576_MOESM1_ESM.zip › S1/IgG/Feb14 0 30 60 90/Blank 2.pdf]

Data File C:\Users\P...s\ChemStation\1\Data\TK_CHO_IgG 2019-02-14 10-52-34\007-1-OPTICHO.D
Sample Name: OPTICHO

===

Acq. Operator : SYSTEM   Seq. Line : 7
Sample Operator : SYSTEM
Acq. Instrument : HPLC-0039   Location : 1
Injection Date : 2/14/2019 11:20:09 AM   Inj : 1
Inj Volume : 100.000 µl
Different Inj Volume from Sample Entry! Actual Inj Volume : 50.000 µl
Sequence File : C:\Users\Public\Documents\ChemStation\1\Data\TK_CHO_IgG 2019-02-14 10-52-34 \TK_CHO_IgG.S
Method : C:\Users\Public\Documents\ChemStation\1\Data\TK_CHO_IgG 2019-02-14 10-52-34 \CHO_IgG_LC.M (Sequence Method)
Last changed : 2/8/2019 3:17:23 PM by SYSTEM

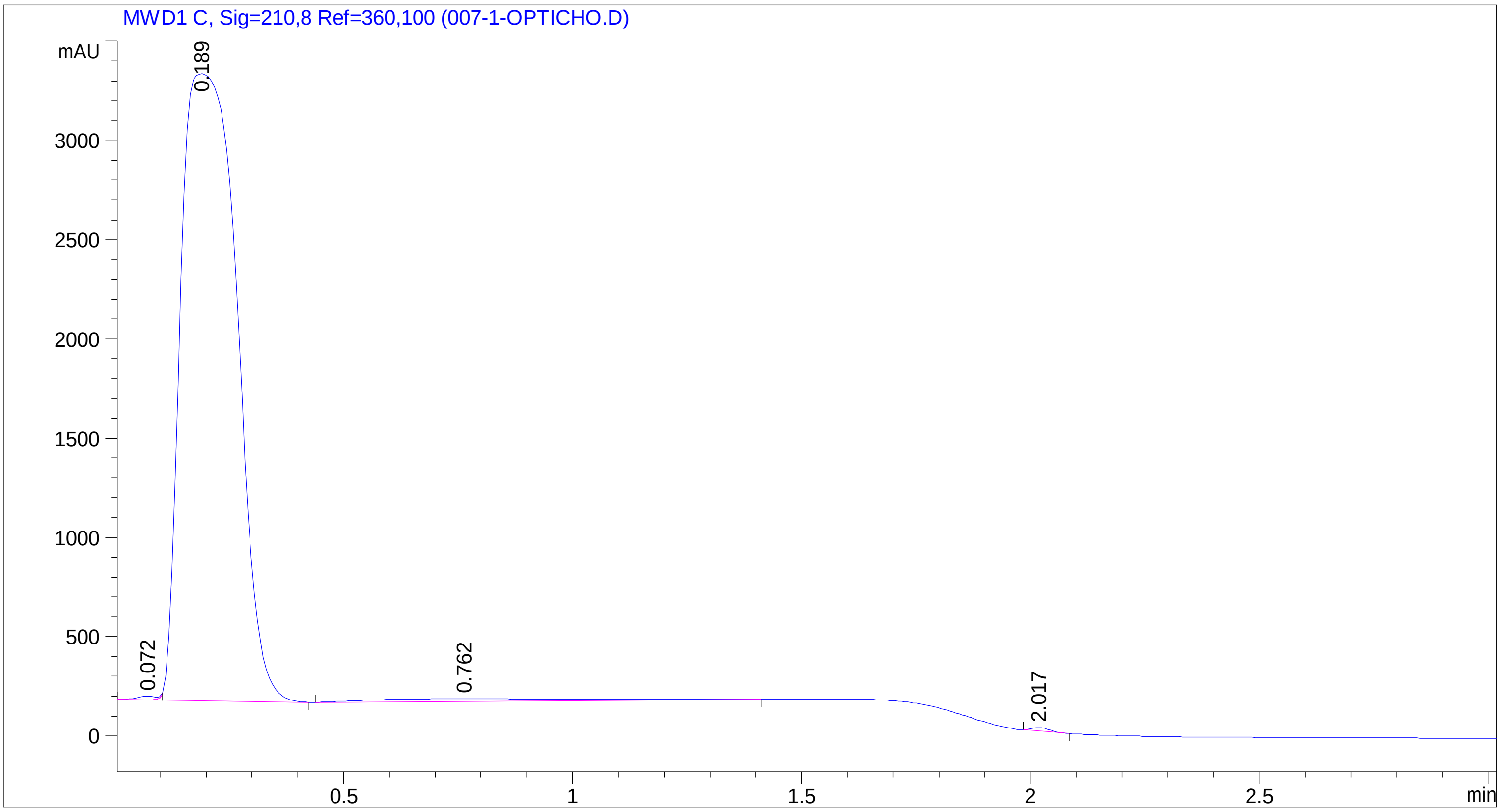

===

Area Percent Report

===

Sorted By : Signal
Multiplier : 1.0000
Dilution : 1.0000
Use Multiplier & Dilution Factor with ISTDs

Signal 1: MWD1 C, Sig=210,8 Ref=360,100

Peak #	RetTime [min]	Type	Width [min]	Area [mAU*s]	Height [mAU]	Area %
1	0.072	BV E	0.0447	48.67513	17.49536	0.1793
2	0.189	VB R	0.1405	2.66572e4	3157.96899	98.2179
3	0.762	BB	0.4893	388.23141	11.75437	1.4304
4	2.017	BBA	0.0432	46.76895	17.48669	0.1723

Totals : 2.71409e4 3204.70541

===

*** End of Report ***
